# A suicide attempt by intoxication with *Taxus baccata* leaves and ultra-fast liquid chromatography-electrospray ionization-tandem mass spectrometry, analysis of patient serum and different plant samples: case report

**DOI:** 10.1186/s40360-016-0078-5

**Published:** 2016-08-30

**Authors:** Małgorzata Kobusiak-Prokopowicz, Anna Marciniak, Sylwester Ślusarczyk, Krzysztof Ściborski, Aneta Stachurska, Andrzej Mysiak, Adam Matkowski

**Affiliations:** 1 Department of Cardiology, Medical University of Wroclaw, ul. Borowska 213, Wroclaw, 50-556 Poland; 2 Department of Pharmaceutical Biology and Botany, Medical University of Wroclaw, Wroclaw, Poland; 3 Department of Biochemistry, IUNG Institute of Plant Cultivation and Soil Science, Pulawy, Poland

**Keywords:** Taxus, Yew alkaloids, Liquid chromatography, Mass spectrometry, Acute heart failure

## Abstract

**Background:**

Taxus (yew) is one of the most frequently reported plants causing potentially fatal outcome when taken incidentally or for suicidal reasons. A fast and reliable method of detection of poisonous compounds or their metabolites is critical in life-saving procedures in cases of yew ingestion. Previously, several chromatographic analytical procedures have been described usually taking longer than one hour of total analysis time.

**Case presentation:**

In this report we describe a suicide case study and an ad hoc developed fast method of detection and quantitation of 3,5-dimethoxyphenol – the main taxane metabolite in the blood plasma from the patient as well as the determination of major taxine components in the plant material (*Taxus baccata*). At present, there is no reasonable alternative for mass spectrometry that could match its high sensitivity and accuracy, and Multiple Reaction Monitoring could be adequate and useful mass spectrometry technique in analyzing and identification of plants material compounds that cause severe poisoning in humans. In the reported case, intensive cardiac care together with the astuteness of the treating physicians not only saved the patient’s life, but also allowed for his complete recovery and return to work.

**Conclusions:**

The development of ultra fast liquid chromatography tandem mass spectrometry UFLC-MS/MS method provides a fast means to confirm yew alkaloids and their metabolite in various material. The applied analytical procedure allows early detection of main metabolite in patient material as well as comparing to those extracted from the plant. In our study, the taxanes remained undetected, probably due to the time elapsing from the patient admittance and collection of plasma. In cases like those reported in this study, retaining the gastric material should be obligatory to confirm the ingestion of yew. The possibility of using this approach in detection of native taxine compounds in human plasma remains to be verified.

## Background

The toxicity of Taxus spp. (yew) evergreen gymnosperm shrubs or trees has been known since antiquity. Regrettably, many Internet resources describe the use of various parts of the yew tree as a classical way of committing suicide [1]. The European yew (*Taxus baccata* L.) is commonly used in decorative landscaping, especially in hedges. It is widely planted and easily available in parks, streets, and gardens in the city of Wroclaw (southwestern Poland). The major active compounds of yew (approximately 30 % of the total alkaloid fraction) include a mixture of compounds called taxines, including the main alkaloids taxine B and isotaxine B (1.2 % dry weight in leaves) [2]. Taxoid levels in the plant vary seasonally, with the highest concentration found in January [3]. Taxane-type diterpenoid alkaloids (e.g., taxine A and B, isotaxine B, paclitaxel, and taxol B) and glycosides (e.g., taxicatine) seem to be responsible for the toxicity of yew leaves, causing typical symptoms of nausea, vomiting, diffuse abdominal pain, tachycardia (initially), and convulsions, followed by bradycardia and respiratory muscle paralysis [4]. If there is no immediate medical intervention, the poisoning is usually fatal. A lethal dose for an adult person has been reported to be 50 g of fresh yew leaves, equaling 250 mg of taxine alkaloids or 3 mg of taxine per kilogram of body weight [5].

In this report, we describe a suicide case study and an ad hoc developed fast method of detection and quantitation of 3,5-dimethoxyphenol (3,5-DMP) – the main taxane metabolite in the blood plasma from the patient as well as the determination of major taxine components in the plant material.

## Case presentation

### Case report

A 46-year-old man with no remarkable past medical history was brought to the emergency department of the University Clinical Hospital in Wrocław. As reported by the emergency medical service physician, the man was found lying under a park bench. He was obtunded, reported nausea and vomiting, but denied any chest pain. The physician noted anisocoria (left > right).

**Fig. 1:**
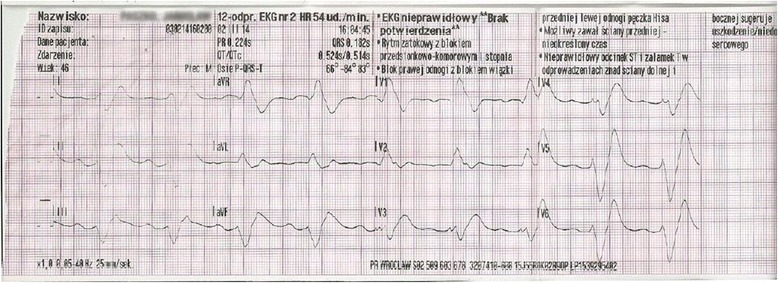
Electrocardiogram at 16:04:29, Mar 02, 2014. Sinus bradycardia at 55 bpm. Left axis deviation. Intraventricular conduction disturbances. First degree atrioventricular block (PR interval 440 ms). Right bundle branch block (QRS duration 280 ms). QTc prolongation (479 ms according to the Bazett formula)

**Fig. 2:**
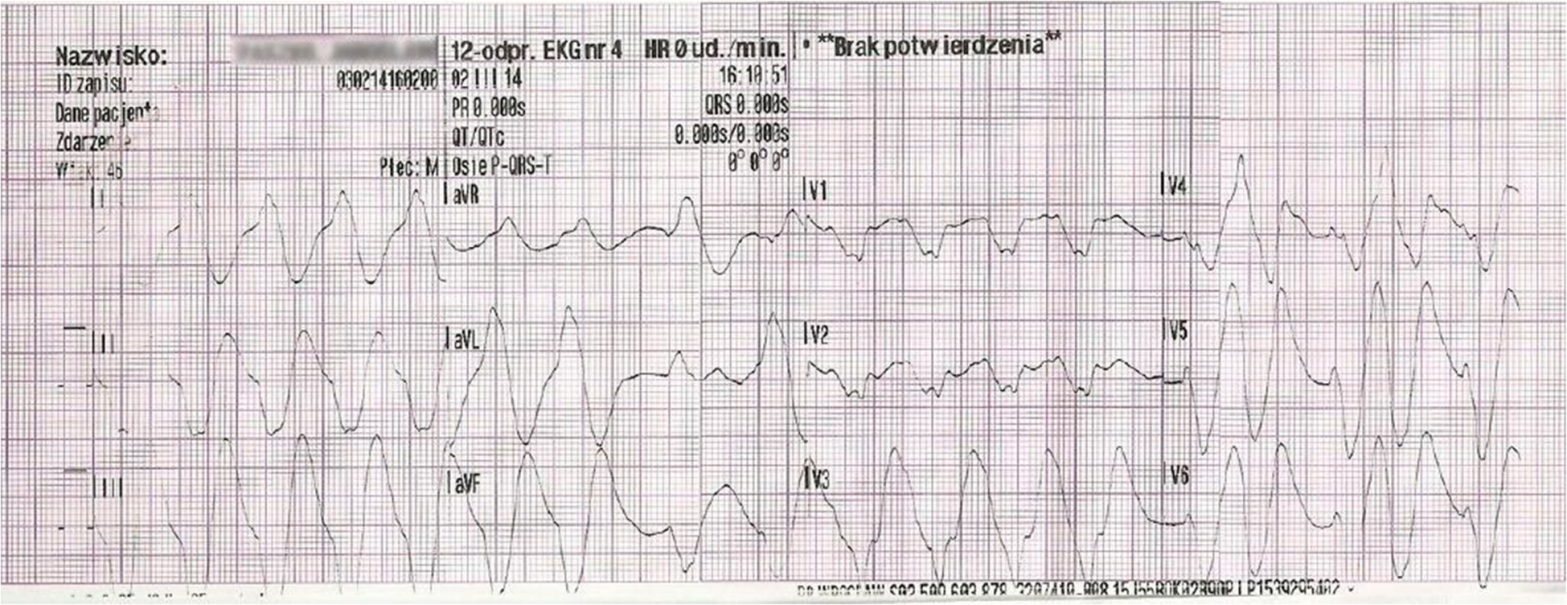
Electrocardiogram at 16:10:51, Mar 02, 2014. Ventricular tachycardia at 120 bpm

**Fig. 3:**
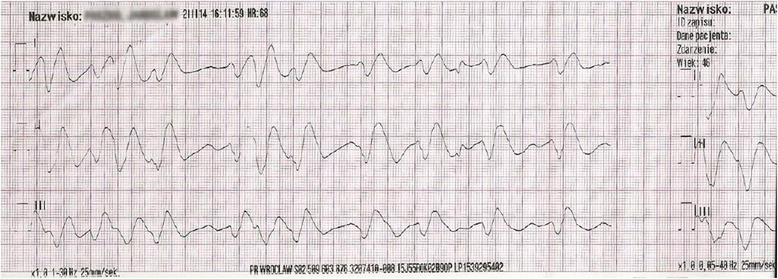
Electrocardiogram at 16:11:59, Mar 02, 2014. Polymorphic ventricular tachycardia (torsade de pointes) at approximately 150 bpm

The initial electrocardiogram (ECG) recorded at 04:04 PM showed sinus bradycardia with a rate of 55 beats per minute (bpm), first degree atrioventricular block, widened QRS complexes, and ST elevation in leads II, III, aVF, and V3–V6 (Fig. 1). Suspicion of an acute coronary syndrome (ACS) was raised. Within 5 min, ventricular tachycardia developed (Fig. 2), followed by torsade de pointes with a rate of 150 bpm (Fig. 3). At 04:28 PM, cardiac arrest due to bradycardia/asystole occurred. Sinus rhythm returned after a short application of external cardiac massage. While the patient was in the emergency department, basic and advanced life support was initiated several times (with 1 mg adrenalin injection three times) due to cardiac arrest caused by bradycardia and asystole. Intravenous dopamine infusion was started and access to the right jugular vein was obtained. Emergency coronary angiography showed no significant coronary lesions. A consulting neurologist ordered computed tomography of the brain which also showed no significant abnormalities.

Abnormal laboratory test results included elevated levels of dimer D (1.19 μg/mL), gamma-glutamyl transpeptidase (119 U/L), aspartate transaminase (238 U/L), alanine transaminase (172 U/L), urea (68 mg/dL), creatinine (1.40 mg/dL), plasma glucose (323 mg/dL), and hypokalemia (3.3 mmol/L). Troponin I level was normal. Arterial blood gases showed metabolic acidosis.

On admission to the Intensive Cardiac Care Unit, the patient was severely ill, sedated with midazolam and fentanyl, intubated, ventilated, and treated with intravenous dopamine infusion. Bedside echocardiography showed dilated vena cava inferior (28 mm) without respiratory variability, paradoxical interventricular septal motion (D-sign), and impaired left ventricular systolic function with an ejection fraction of 40 %. Pulmonary embolism was suspected and pulmonary computed angiotomography was performed which showed no evidence of embolism.

At 11:05 PM, cardiac arrest due to pulseless electrical activity occurred and resuscitation was started immediately. ECG showed recurrent torsade de pointes, requiring several defibrillations and administration of magnesium and antiarrhythmic drugs, and periods of bradycardia. The overall duration of resuscitation attempts was 1.5 h. The patient was treated with intravenous dopamine (200 μg/50 mL 0.9 % NaCl, rate 15 mL/h), norepinephrine (8 mg/50 mL 0.9 % NaCl, rate 30 mL/h), and dobutamine (250 μg/50 mL 0.9 % NaCl, rate 5 mL/h) infusions and adrenaline in boluses (6 × 1 mg during resuscitation) followed by infusion (2 mg/50 mL 0.9 % NaCl, rate 10 mL/h) beginning at 02:30 AM. The patient also received amiodarone, lidocaine, bicarbonates, and intravenous fluids (1000 mL 0.9 % NaCl, yielding 2700 mL of urine). Due to ineffective antiarrhythmic drug therapy and bradycardia, a transvenous pacing lead was inserted, allowing effective pacing and resolution of shock.

Despite treatment with amiodarone, magnesium, and lidocaine, correction of electrolyte disturbance, and several dozens of defibrillation attempts, the arrhythmia underlying cardiac arrest persisted. Only after overdrive pacing was established using a transvenous pacing lead could arrhythmia be controlled and the patient’s condition stabilized.

As the underlying cause of the patient’s clinical condition remained unknown, the patient’s family was contacted. The family reported that 2–3 weeks before admission to the Department of Cardiology, the patient was consulted by a psychiatrist due to a suspected suicide attempt. He had numerous financial debts resulting from his addiction to internet gambling. The toxicology screen and serum digoxin assay on admission were negative. The family was asked to search for any toxic substances among the patient’s personal belongings at home and work. A decoction of unknown origin was found in the patient’s work locker and his mobile phone’s internet history included websites discussing toxic plants, including yew (*Taxus baccata*). Due to a suspicion of *Taxus baccata* intoxication (bradycardia leading to asystole, vomiting, orange-pink color of urine), the decoction and patient’s blood sample were sent to the Department of Pharmaceutical Biology and Botany for biochemical analysis.

### Methods of extraction of plant material from *T. baccata*

Fresh leaves (needles) (20 g) of *T. baccata* were harvested from two different locations. The first sample was from the vicinity of where the suicide attempt took place (yew1) and the second one was from the certified collection of the Wroclaw Medical University Botanical Garden where *T. baccata* is cultivated (yew2). The voucher specimens of the two plant samples are stored in the herbarium of the Botanical Garden.

All material was ultra-sonicated (IS-36 ultrasonic bath, Intersonic, Poland) in 200 mL of ethanol (analytical grade, 95 %) for 30 min and soaked for 16 h overnight. The crude extract was filtered, the solvent evaporated, and the residue was reconstituted with 80 mL 1 N HCl and extracted with 80 mL dichloromethane. The aqueous phase was alkalized with ammonia solution (25 %) and extracted with 240 mL dichloromethane (3 × 80 mL). The organic phases were pooled and the solvent evaporated, yielding 80 mg of a white residue (0.4 % of the fresh mass of yew leaves). A methanolic solution of this residue was diluted with mobile phases A and B (50:50, *v*/*v*) for further analysis.

Two mL of the patient’s blood serum sample obtained from the coronary care unit were precipitated by adding 2 mL of acetonitrile, followed by centrifugation at 6000 g for 7 min. The supernatant was then purified by the solid phase extraction method using octadecyl silica bed cartridges (Supelco, USA). All analyzed samples were filtered with a 0.45 μm membrane filter (PTFE, Carl Roth, Germany) before injection into the ultra fast liquid chromatography (UFLC) system.

#### UFLC and MS/MS conditions

The UFLC system consisted of an LC-30ADXR pump, a DGU-20A3 degasser, a SIL-20AXR autosampler, a CTO-10ASVP column heater, and a CBM-20A system controller (Shimadzu Ltd. Japan). Chromatographic separations were performed on a Kinetex C18 100A column (2.1 mm × 100 mm, 2.6 μ) with a security guard column (Phenomenex, U.S.A.).

The mobile phase was composed of acetonitrile-water with 0.1 % (*v*/*v*) formic acid. A gradient program was employed with the mobile phase, combining solvent A (water) and solvent B (acetonitrile) with 0.1 % formic acid as follows: 80 % A (0–0.5 min), 80–40 % A (0.5–7 min), 40–20 % A (7–12 min), 20–5 % A (12–15 min), 5–0 % A (15–16 min), 0 % A (16–17 min), 0–80 % A (17–18 min), 80 % A (18–22 min).

The flow rate was 0.3 mL/min and the injection volume was 1 μL. The column and sample temperatures were maintained at 35 and 4 °C, respectively. All data acquisition and peak integration were performed using LabSolution (5.53 SP2) from Shimadzu Ltd.

Mass spectrometric detection was performed on a Shimadzu LCMS-8030 with an electrospray ionization (ESI). The analytes were determined in the positive ionization mode and quantified by multiple-reaction monitoring (MRM) mode. The parameters and conditions were optimized as follows: capillary voltage of 4.5 kV, desolvation line (DL) temperature of 250 °C, heat block temperature of 400 °C, and nebulizing gas flow and drying gas flow of 3 L/min and 15 L/min, respectively.

**Fig. 4:**
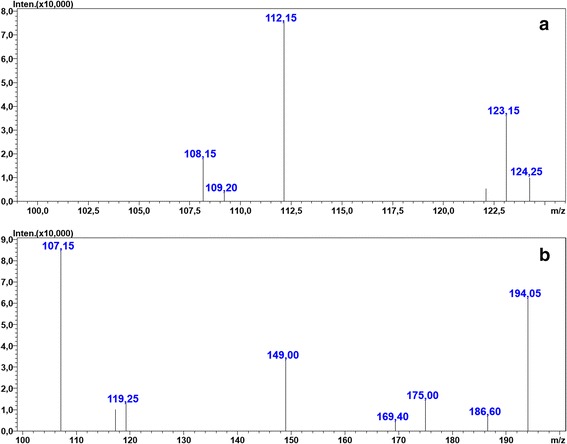
MRM spectra of (**a**) – 3,5-dimethoxyphenol, and (**b**) – taxine B in the patient’s blood sample. Multiple-reaction monitoring (MRM) spectra obtained with triple-quadrupole mass spectrometer of the main compounds from yew found in patient blood sample. **a** 3,5-dimethoxyphenol; **b** taxine B (isotaxine B). Settings of the mass spectrometer are described in the section: *UFLC and MS/MS conditions*

**Fig. 5:**
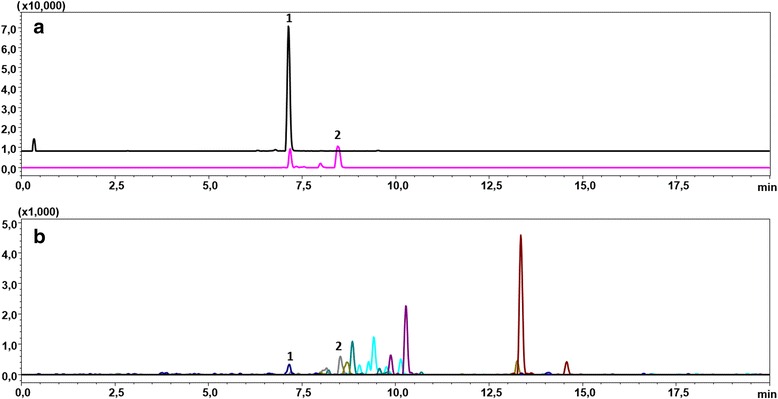
UFLC-MRM chromatograms of blood and yew1 samples showing presence of 3,5-dimethoxyphenol (1) and taxine B (2). Multiple-reaction monitoring (MRM) ultra fast liquid chromatograms (UFLC) of patient blood sample (**a**), and yew1 sample (**b**) showing two main compounds from yew −1: 3,5-dimethoxyphenol; 2: taxine B (isotaxine B). Instrument specifications and method settings are described in the section: *UFLC and MS/MS conditions*

Qualitative confirmation was achieved using UFLC MS/MS analysis with MRM (Table 1). The obtained spectra (Figs. 4 and 5) were compared to the literature data [6, 7].

**Table 1:** Main taxanes found in yew needles and patient blood and their mass spectrometric characteristics

No	Compound name	[M + H]^+^	MRM transitions	CE	Yew 1	Yew 2	Serum
1	Paclitaxel (TaxolA)	854.2	854.2 > 286.1 > 105.0	48	ND	ND	ND
2	10-Deacetyltaxol (DAT)	812.2	812.2 > 286.1 > 105.0	48	ND	ND	ND
3	Baccatin III (BAC III)	587.0	587.0 > 327 > 105.0	65	+	ND	ND
4	10-Deacetylbaccatin III (DAB)	545.0	545.0 > 327 > 105.0	58	ND	ND	ND
5	Cephalomannine (Taxol B)	832.3	832.3 > 509 > 264	20	+	ND	ND
6	3,5-Dimethoxyphenol (3,5-DMP)	155.2	155.2 > 123.1 > 112.1	25	+++	++	+++
7	Monoacetyltaxine (MAT)	568.4	568.4 > 194.1 > 107.2	40	++	+	ND
8	Taxine B/Isotaxine B	584.2	584.2 > 194.1 > 107.2	40	+++	++	+
9	Monohydroxydiacetyltaxine (MHDAT)	626.4	626.4 > 194.2 > 106.8	45	+	+	ND
10	Triacetyltaxine (TAT)	652.3	652.3 > 194.1 > 107.2	45	++	+	ND
11	Monohydroxytriacetyltaxine (MHTAT)	668.4	668.4 > 107.2 > 103.1	48	++	+	ND
12	Taxine A	642.2	642.2 > 194.1 > 106.8	44	+	+	ND
13	2-Deacetyltaxin A	602.2	602.2 > 194.1 > 106.8	35	+	+	ND
14	Taxicatine	317.3	317.3 > 155.2	16	++	+	ND

*CE* energy [eV] of collision-induced dissociation, *MRM* multiple-reaction monitoring (positive ESI), *ND* not detected

The main substance of taxine origin identified in the blood sample (serum) was 3,5-DMP, the aglycone of taxicatine (3,5-dimethoxyphenol glucoside), which is an ingredient of yew leaves. This compound was suggested by Musshof et al. [8] as a convenient marker of poisoning with Taxus baccata leaves. We observed this compound in both yew1 and yew2 samples. Other toxins including taxine A, 2-deacetyltaxine, monoacetyltaxin, taxine B (isotaxine B), monohydroxydiacetyltaxine, triacetyltaxin, and monohydroxytriacetyltaxine were also detected. We did not detect paclitaxel (Taxol A), deacetyltaxol, 10-(DAT), deacetylbaccatin III 10-(DAB) in any of the analyzed samples. These compounds are frequently listed as yew constituents. However, they may not have been extracted from our plant material with the method used.

Nonetheless, the liquid chromatography tandem mass spectrometry (LC-MS/MS) method was useful for fast and reliable detection of yew toxin residues in the blood sample.

The main taxine ingredients are presented in Table 1.

## Case report - continued

The patient’s clinical condition improved gradually over the next few days. At the second day of hospital stay, the patient was weaned from mechanical ventilation and extubated. Follow-up arterial blood gases were normal. Catecholamine infusions were also weaned and the transvenous pacing lead was removed. ECG showed junctional escape rhythm followed by normal sinus rhythm at a rate of 75 bpm. Laboratory testing showed gradual normalization of kidney and liver function. Evidence of disseminated intravascular coagulation was also seen. Following a hematology consultation, a therapeutic dose of low-molecular-weight heparin was started, leading to a gradual improvement of coagulation parameters. Follow-up echocardiography showed resolution of segmental wall motion abnormalities with a normal left ventricular ejection fraction of 70 %. Twenty-four-hour Holter ECG monitoring showed no significant tachy- or bradyarrhythmia.

Upon full return of consciousness, the patient admitted consuming yew leaves, initially a few twigs in an unprocessed form (in a sandwich), and later a decoction made from several more twigs.

A consulting neurologist found no clear evidence of central nervous system damage. A consulting psychiatrist noted slight memory impairment in the period following the suicide attempt and decreased mood. During the hospitalization, the patient did not reveal active suicidal thoughts or tendencies. Inpatient psychiatric treatment and initiation of a selective serotonin reuptake inhibitor (sertraline) was recommended, along with complete abstinence and addiction treatment. The patient was discharged home in a stable general condition and referred for psychiatric treatment.

## Discussion

The toxic effects of the yew tree (*Taxus baccata*) and its leaves have been known since ancient times. The seeds are generally responsible for accidental intoxications in childhood, whereas the bark and the leaves are mainly used for homicidal or suicidal attempts. High levels of conjugated metabolites found in urine highlight the critical role that conjugation in the liver plays in eliminating taxines and increasing the probability of a patient’s survival.

Despite severe toxicity, fatal poisonings with Taxus spp. are rarely reported [3, 4]. Most cases have been described in young subjects ingesting yew needles with suicidal intentions [5]. The toxicity of Taxus arises from its alkaloids taxines A and B which are present in all parts of the plant except for the red arils. Taxines block sodium and calcium channels, preferentially in cardiomyocytes, thus causing conduction abnormalities [4, 6]. Treatment of Taxus poisoning is supportive [7, 8]. Our patient presented with typical symptoms of Taxus intoxication, i.e., broad QRS complex brady/tachyarrhythmia with atrioventricular block, depressed consciousness, and respiratory distress [2–4, 6–8]. The ECG pattern was complex and difficult to interpret, described in the literature as the “dying heart” [9]. Multiorgan failure in our patient did not require renal replacement therapy, which was also reported in the literature [10].

Polymorphic ventricular tachycardia, which usually leads to death unless advanced life support measures are initiated, is typically caused by atrioventricular blocks, QT interval prolongation, hypokalemia, or intoxication with drugs, poisonings and toxic substances, as in the reported patient. Notably, the patient suffered from depression and an attempted suicide was suspected (based on information obtained from the family), as was intoxication with an unknown substance. Further signs of yew intoxication are nausea, vomiting, abdominal pain, and seizures, all of which could not be ascertained in the patient due to reduced consciousness. In line with earlier reports [11], our patient informed himself about the toxicity of Taxus baccata on various web sites.

The European Society of Cardiology guidelines on the management of ventricular arrhythmia and prevention of sudden cardiac death recommend withdrawal of drugs and/or toxins (*T. baccata* in this case, which was initially not known to the treating physicians) that might cause arrhythmia, correcting electrolyte disturbances, and short-term or chronic pacing in patients with torsade de pointes due to conduction disturbances or symptomatic bradycardia [12]. Intravenous administration of magnesium sulfate, which is indicated in patients with long QT syndrome, may be ineffective in patients with normal QT intervals. Short- or long-term pacing is reasonable in patients with recurrent torsade de pointes, along with potassium administration, beta-blocker treatment (following insertion of a pacing lead), and possibly administration of lidocaine [13, 14].

In this particular case, the access to fast and reliable analytical procedure would facilitate the rational therapeutic approach. Hence, our method based on fast multiple reaction monitoring liquid chromatography tandem mass spectrometry (MRM-LC-MS/MS) detection allows to complete the analysis in less than 45 min, including sample preparation. This approach would be even more advantageous in more severe poisoning and could be crucial for life saving decision making of a critical care team.

Several analytical procedures are available in the toxicological and analytical literature, based on liquid or gas chromatography, usually employing mas spectrometry detection of taxanes or their metabolites. The most accurate and reliable methods are based on hyphenated techniques such as gas or liquid chromatography coupled with mass selective detectors with best results obtained with tandem mass spectrometry [2, 7, 15]. MRM mode used in our study is recommended for its higher specificity and ability to distinguish a higher number of compounds during the single run.

Using less expensive high pressure liquid chromatography (HPLC) equipment with photometric detection requires co-chromatography with authentic standards for each compound which is impractical for difficult to purchase or synthesize, non-typical metabolites and the methods based on retention time are also prone to misidentification [15].

At present, there is no reasonable alternative for mass spectrometry that could match its high sensitivity and accuracy. Other methods of detection, such as vibrational spectroscopy or nuclear magnetic resonance (NMR) are less sensitive or do not provide insight into the diversity of specific taxane metabolites and are rather applied to characterize plant material than body fluids [16, 17]. However, along with the technological progress in the instrumentation and metabolomic data processing, these methods could become more practical, due to a relatively simple procedures of sample preparation and comparability of results between different instruments and analysis conditions [18]. Highly sensitive immunological methods such as ELISA have been also reported as suitable for detecting taxanes in physiological fluids. These methods allow for analysis of many samples simultaneously, but their disadvantage is usually too narrow specificity and varying antibodies affinity, leaving potentially diagnostic metabolites undetected or misestimated [19].

On the other hand, gas chromatography mass spectrometry (GC-MS) is a well established and reliable method, but requires more laborious sample preparation but reasonable separation time if targeting just 3,5-DMP [20]. The advantage over liquid chromatography mass spectrometry (LC-MS) is the standardization of methodology and availability of vast libraries of spectra that allows identification of compounds without having an authentic standard for each.

In the reported case, intensive cardiac care together with the astuteness of the treating physicians not only saved the patient’s life, but also allowed for his complete recovery and return to work. The applied analytical procedure is fast and allows early detection of main metabolite in patient material as well as comparing to those extracted from the plant. In a complex and detailed study of eight intoxication cases [2] utilized a qTRAP mass detector and allowed detection and quantitation of several taxanes along with 3,5-DMP. In our study, the taxanes remained undetected, probably due to the time elapsing from the patient admittance and collection of plasma. In such cases, retaining the gastric material should be obligatory to confirm the ingestion of yew. The possibility of using this approach in detection of native taxine compounds in human plasma remains to be verified.

## Conclusions

The development of ultra fast liquid chromatography tandem mass spectrometry UFLC-MS/MS method provides a fast means to confirm yew alkaloids and their metabolite in various material. The applied analytical procedure allows early detection of main metabolite in patient material as well as comparing to those extracted from the plant. In our study, the taxanes remained undetected, probably due to the time elapsing from the patient admittance and collection of plasma. In cases like those reported in this study, retaining the gastric material should be obligatory to confirm the ingestion of yew. The possibility of using this approach in detection of native taxine compounds in human plasma remains to be verified.

## Abbreviations

3,5-DMP, 3,5-dimethoxyphenol; ACS, acute coronary syndrome; bpm, beats per minute; DL, desolvation line; ECG, electrocardiogram; ESI, electrospray ionization; GC-MS, gas chromatography mass spectrometry; HPLC, high pressure liquid chromatography; LC-MS, liquid chromatography mass spectrometry; LC-MS/MS, liquid chromatography tandem mass spectrometry; MRM, multiple-reaction monitoring; MRM-LC-MS/MS, multiple reaction monitoring liquid chromatography tandem mass spectrometry; NMR, nuclear magnetic resonance; UFLC, ultra fast liquid chromatography.
